# (*Z*)-2-(2-Isopropyl-5-methyl­phen­oxy)-*N*′-(2-oxoindolin-3-yl­idene)acetohydrazide

**DOI:** 10.1107/S1600536810019008

**Published:** 2010-06-09

**Authors:** Chetan M. Zade, Umesh D. Pete, Amol G. Dikundwar, Ratnamala S. Bendre

**Affiliations:** a School of Chemical Sciences, North Maharashtra University, Jalgaon 425 001, India; b Solid State and Structural Chemistry Unit, Indian Institute of Science, Bangalore 560 012, Karnataka, India

## Abstract

In the title Mannich base, C_20_H_21_N_3_O_3_, an isatin derivative of thymol, the O—CH_2_—C(=O)–N(H)—N fragment connect­ing the aromatic and fused-ring systems is approximately planar, with the N—N single bond in a *Z* configuration. The amino H atom of this N—N fragment is intra­molecularly hydrogen bonded to the carbonyl O atom of the indolinone fused ring as well as to the phen­oxy O atom of the aromatic ring. The amino H atom of the indoline fused ring forms a hydrogen bond with the double-bond O atom of an adjacent mol­ecule, this hydrogen bond giving rise to a linear chain motif.

## Related literature

For the synthesis, see: Khan *et al.* (2007 ▶); Nargud *et al.* (1996 ▶); Shah *et al.* (1996 ▶). For related structures, see: Butcher *et al.* (2005 ▶, 2007 ▶).
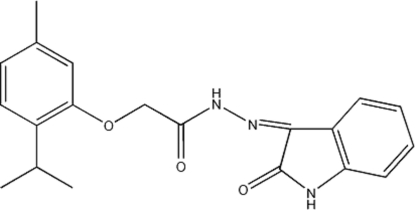


## Experimental

### 

#### Crystal data

C_20_H_21_N_3_O_3_
*M* *_r_* = 351.40
Triclinic, 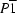

*a* = 7.6890 (4) Å
*b* = 8.2206 (5) Å
*c* = 15.3588 (9) Å
α = 81.423 (5)°
β = 86.843 (5)°
γ = 67.992 (5)°
*V* = 890.00 (9) Å^3^
*Z* = 2
Mo *K*α radiation
μ = 0.09 mm^−1^
*T* = 100 K
0.40 × 0.20 × 0.20 mm

#### Data collection

Oxford Xcalibur Eos (Mova) CCD detector diffractometer
Absorption correction: multi-scan (*CrysAlis RED*; Oxford Diffraction, 2009 ▶) *T*_min_ = 0.965, *T*_max_ = 0.982
19920 measured reflections
3491 independent reflections
2569 reflections with *I* > 2σ(*I*)
*R*_int_ = 0.049

#### Refinement

*R*[*F* ^2^ > 2σ(*F* ^2^)] = 0.047
*wR*(*F* ^2^) = 0.126
*S* = 1.08
3491 reflections
246 parameters
H atoms treated by a mixture of independent and constrained refinement
Δρ_max_ = 0.54 e Å^−3^
Δρ_min_ = −0.42 e Å^−3^

### 

Data collection: *CrysAlis PRO CCD* (Oxford Diffraction, 2009 ▶); cell refinement: *CrysAlis PRO CCD*; data reduction: *CrysAlis PRO RED* (Oxford Diffraction, 2009 ▶); program(s) used to solve structure: *SHELXS97* (Sheldrick, 2008 ▶); program(s) used to refine structure: *SHELXL97* (Sheldrick, 2008 ▶); molecular graphics: *ORTEP-3* (Farrugia, 1997 ▶) and *CAMERON* (Watkin *et al.*, 1996 ▶); software used to prepare material for publication: *WinGX* (Farrugia, 1999 ▶).

## Supplementary Material

Crystal structure: contains datablocks global, I. DOI: 10.1107/S1600536810019008/ng2756sup1.cif

Structure factors: contains datablocks I. DOI: 10.1107/S1600536810019008/ng2756Isup2.hkl

Additional supplementary materials:  crystallographic information; 3D view; checkCIF report

## Figures and Tables

**Table 1:** Hydrogen-bond geometry (Å, °)

*D*—H⋯*A*	*D*—H	H⋯*A*	*D*⋯*A*	*D*—H⋯*A*
N3—H1⋯O1	0.91 (2)	2.06 (2)	2.768 (2)	134.1 (19)
N3—H1⋯O3	0.91 (2)	2.17 (2)	2.574 (2)	106.1 (17)
N1—H2⋯O2^i^	0.91 (2)	1.93 (2)	2.8174 (19)	164 (2)

Symmetry code: (i) 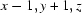.

**Fig. 1.**
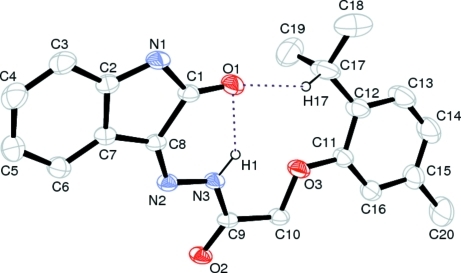
Molecular structure of (I) with intramolecular hydrogen bonds. Displacement ellipsoids drawn at the 50% probability level, the H atoms involved in intramolecular hydrogen bonds are shown as small spheres of arbitrary radius.

**Fig. 2.**
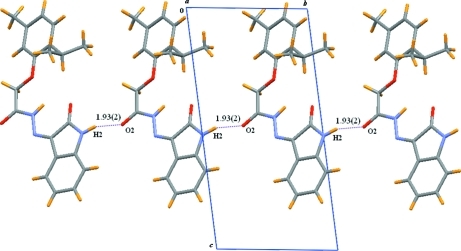
Packing diagram of (I) viewed down the a axis. The dotted lines indicate intermolecular N—H···O interactions.

## supplementary crystallographic information

### Comment

Several natural phenol derivatives such as carvacrol [5-isopropyl-2-methylphenol], thymol [5-methyl-2-(1-methylethyl) phenol] and eugenol [2-methoxy-4-(2-propenyl)phenol] and their structural analogues show antimicrobial effects. Structural modification of these monoterpenoids has been carried out to improve their biological activity. The crystal structures of compounds containing thymol moiety have been reported by us (Butcher *et al.* 2007 and Butcher & Bendre 2005). The title compound, (*Z*)-2-(2-isopropyl-5-methylphenoxy) -*N*'-(2-oxoindolin-3-ylidene)acetohydrazide was synthesized by considering the importance of phenolic monoterpenoid thymol, 2,3-dioxindole derivatives and its Mannich bases in the enhancement of the biological activity of these compounds. The molecular conformation depicts an interplanar angle of 11.48 between the two rings in the molecule which results from both N—H···O and C—H···O intramolecular hydrogen bonds (Table 1, Fig.1). The molecules are packed mainly by intermolecular N—H···O hydrogen bonds across the centre of inversion which form molecular chains along the crystallographic b-direction. The packing is further stabilized by weak C—H···pi and pi···pi interactions.

### Experimental

The title compound was synthesized from a mixture of 2-(2-isopropyl-5-methylphenoxy)acetohydrazide and isatin. Equimolar quantities of 2-(2-isopropyl-5-methylphenoxy)acetohydrazide (Nargud *et al.* 1996 and Shah *et al.* 1996) and isatin (0.02 mole) in 50 ml of dioxan were taken in a 100 ml round bottom flask. To this mixture 5 ml of glacial acetic acid was added. The reaction mixture was refluxed for about 2 hours and then cooled. A solid separated was filtered off with suction to isolate the product (82 % yield) and recrystallized in ethanol, yellow crystals suitable for X-ray diffraction were obtained.

IR (Nujol, cm^-1^):3400-3300 (N—H of amide), 3209 (N—H of ring), 1715 (C=O of isatin ring), 1686 (C=O of acyclic amide) and 1601 (C=N).

1H NMR (DMSO-d6, ppm): 1.17 (d, 12H, gem CH3), 2.25 (s, 6H, Ar—CH3), 3.56 (heptet, 1H, C—H), 4.83 (s, 2H, OCH2), 6.80-7.59 (m, 7H, Ar—H), 11.32 (s, 1H, N—H of amide linkage) and 13.55 (s, 1H, N—H of isatin ring).

### Refinement

All H atoms were positioned geometrically, (C—H = 0.93 Å, N—H = 0.86 Å) and refined using a riding model with *U*_iso_(H)= 1.2 U_eq_(C, N) except the two on nitrogen atoms which were located and refined isotropically.

### Crystal data

**Table:**

C_20_H_21_N_3_O_3_	*Z* = 2
*M**_r_* = 351.40	*F*(000) = 372
Triclinic, *P*1	*D*_x_ = 1.311 Mg m^−^^3^
Hall symbol: -P 1	Mo *K*α radiation, λ = 0.71073 Å
*a* = 7.6890 (4) Å	Cell parameters from 19920 reflections
*b* = 8.2206 (5) Å	θ = 2.7–26.0°
*c* = 15.3588 (9) Å	µ = 0.09 mm^−^^1^
α = 81.423 (5)°	*T* = 100 K
β = 86.843 (5)°	Block, yellow
γ = 67.992 (5)°	0.40 × 0.20 × 0.20 mm
*V* = 890.00 (9) Å^3^	

### Data collection

**Table:**

Oxford Xcalibur Eos(Nova) CCD detector diffractometer	3491 independent reflections
Radiation source: Enhance (Mo) X-ray Source	2569 reflections with *I* > 2σ(*I*)
graphite	*R*_int_ = 0.049
ω scans	θ_max_ = 26.0°, θ_min_ = 2.7°
Absorption correction: multi-scan (*CrysAlis RED*; Oxford Diffraction, 2009)	*h* = −9→9
*T*_min_ = 0.965, *T*_max_ = 0.982	*k* = −10→10
19920 measured reflections	*l* = −18→18

### Refinement

**Table:**

Refinement on *F*^2^	Primary atom site location: structure-invariant direct methods
Least-squares matrix: full	Secondary atom site location: difference Fourier map
*R*[*F*^2^ > 2σ(*F*^2^)] = 0.047	Hydrogen site location: inferred from neighbouring sites
*wR*(*F*^2^) = 0.126	H atoms treated by a mixture of independent and constrained refinement
*S* = 1.08	*w* = 1/[σ^2^(*F*_o_^2^) + (0.0676*P*)^2^ + 0.143*P*] where *P* = (*F*_o_^2^ + 2*F*_c_^2^)/3
3491 reflections	(Δ/σ)_max_ = 0.001
246 parameters	Δρ_max_ = 0.54 e Å^−^^3^
0 restraints	Δρ_min_ = −0.42 e Å^−^^3^

### Special details

**Table:**

Geometry. All esds (except the esd in the dihedral angle between two l.s. planes) are estimated using the full covariance matrix. The cell esds are taken into account individually in the estimation of esds in distances, angles and torsion angles; correlations between esds in cell parameters are only used when they are defined by crystal symmetry. An approximate (isotropic) treatment of cell esds is used for estimating esds involving l.s. planes.
Refinement. Refinement of *F*^2^ against ALL reflections. The weighted *R*-factor wR and goodness of fit *S* are based on *F*^2^, conventional *R*-factors *R* are based on *F*, with *F* set to zero for negative *F*^2^. The threshold expression of *F*^2^ > σ(*F*^2^) is used only for calculating *R*-factors(gt) etc. and is not relevant to the choice of reflections for refinement. *R*-factors based on *F*^2^ are statistically about twice as large as those based on *F*, and *R*- factors based on ALL data will be even larger.

### Fractional atomic coordinates and isotropic or equivalent isotropic displacement parameters (Å^2^)

**Table:**

	*x*	*y*	*z*	*U*_iso_*/*U*_eq_	
O2	0.42649 (16)	0.34876 (14)	0.48742 (8)	0.0194 (3)	
O3	0.31459 (17)	0.65223 (16)	0.29166 (8)	0.0259 (3)	
O1	−0.09186 (17)	0.94154 (16)	0.40232 (8)	0.0241 (3)	
N3	0.19339 (19)	0.61754 (19)	0.45023 (10)	0.0180 (3)	
N1	−0.2913 (2)	1.00881 (19)	0.52175 (10)	0.0198 (3)	
C8	−0.0373 (2)	0.7470 (2)	0.54375 (12)	0.0173 (4)	
N2	0.11373 (19)	0.61305 (18)	0.53194 (10)	0.0178 (3)	
C7	−0.1447 (2)	0.7700 (2)	0.62514 (12)	0.0182 (4)	
C9	0.3539 (2)	0.4815 (2)	0.43422 (12)	0.0178 (4)	
C10	0.4428 (2)	0.5057 (2)	0.34560 (11)	0.0195 (4)	
H10A	0.5569	0.5264	0.3529	0.023*	
H10B	0.4750	0.3995	0.3180	0.023*	
C1	−0.1395 (2)	0.9084 (2)	0.47827 (12)	0.0185 (4)	
C11	0.3648 (2)	0.6956 (2)	0.20616 (12)	0.0223 (4)	
C2	−0.2984 (2)	0.9305 (2)	0.60903 (12)	0.0186 (4)	
C16	0.5382 (3)	0.6086 (2)	0.17067 (13)	0.0265 (4)	
H16	0.6275	0.5142	0.2047	0.032*	
C12	0.2263 (3)	0.8408 (2)	0.15720 (13)	0.0257 (4)	
C5	−0.2538 (3)	0.7256 (2)	0.77154 (13)	0.0258 (4)	
H5	−0.2414	0.6574	0.8266	0.031*	
C6	−0.1212 (2)	0.6671 (2)	0.70683 (12)	0.0219 (4)	
H6	−0.0189	0.5610	0.7180	0.026*	
C4	−0.4055 (3)	0.8859 (3)	0.75476 (13)	0.0261 (4)	
H4	−0.4922	0.9235	0.7992	0.031*	
C3	−0.4304 (2)	0.9911 (2)	0.67309 (12)	0.0241 (4)	
H3	−0.5319	1.0979	0.6621	0.029*	
C13	0.2727 (3)	0.8916 (3)	0.07197 (13)	0.0313 (5)	
H13	0.1852	0.9876	0.0381	0.038*	
C17	0.0377 (3)	0.9323 (3)	0.19774 (14)	0.0366 (5)	
H17	0.0594	0.9413	0.2588	0.044*	
C14	0.4449 (3)	0.8043 (3)	0.03569 (14)	0.0351 (5)	
H14	0.4705	0.8420	−0.0220	0.042*	
C15	0.5802 (3)	0.6616 (3)	0.08384 (13)	0.0322 (5)	
C18	−0.0732 (3)	1.1179 (3)	0.15283 (15)	0.0393 (6)	
H18A	0.0036	1.1879	0.1478	0.059*	
H18B	−0.1826	1.1715	0.1871	0.059*	
H18C	−0.1107	1.1117	0.0952	0.059*	
C20	0.7679 (3)	0.5643 (3)	0.04370 (15)	0.0485 (7)	
H20A	0.7589	0.4725	0.0143	0.073*	
H20B	0.8610	0.5126	0.0893	0.073*	
H20C	0.8030	0.6459	0.0020	0.073*	
C19	−0.0803 (3)	0.8207 (3)	0.1998 (2)	0.0737 (11)	
H19A	−0.1085	0.8139	0.1406	0.110*	
H19B	−0.1949	0.8736	0.2308	0.110*	
H19C	−0.0124	0.7037	0.2292	0.110*	
H2	−0.376 (3)	1.117 (3)	0.4998 (15)	0.044 (7)*	
H1	0.141 (3)	0.711 (3)	0.4077 (14)	0.031 (6)*	

### Atomic displacement parameters (Å^2^)

**Table:**

	*U*^11^	*U*^22^	*U*^33^	*U*^12^	*U*^13^	*U*^23^
O2	0.0189 (6)	0.0146 (6)	0.0197 (7)	−0.0008 (5)	−0.0029 (5)	−0.0007 (5)
O3	0.0212 (7)	0.0253 (7)	0.0201 (7)	0.0012 (5)	0.0010 (5)	0.0045 (5)
O1	0.0221 (7)	0.0207 (7)	0.0222 (7)	−0.0023 (5)	0.0023 (5)	0.0033 (5)
N3	0.0170 (7)	0.0146 (7)	0.0179 (8)	−0.0017 (6)	−0.0005 (6)	−0.0002 (6)
N1	0.0172 (7)	0.0138 (7)	0.0222 (8)	0.0006 (6)	−0.0012 (6)	−0.0001 (6)
C8	0.0150 (8)	0.0157 (8)	0.0208 (10)	−0.0049 (7)	−0.0018 (7)	−0.0024 (7)
N2	0.0159 (7)	0.0165 (7)	0.0197 (8)	−0.0046 (6)	0.0004 (6)	−0.0026 (6)
C7	0.0167 (8)	0.0166 (9)	0.0210 (10)	−0.0052 (7)	−0.0005 (7)	−0.0042 (7)
C9	0.0147 (8)	0.0152 (9)	0.0229 (10)	−0.0040 (7)	−0.0036 (7)	−0.0036 (7)
C10	0.0174 (8)	0.0168 (9)	0.0195 (10)	−0.0017 (7)	−0.0013 (7)	−0.0003 (7)
C1	0.0165 (8)	0.0152 (8)	0.0235 (10)	−0.0055 (7)	−0.0023 (7)	−0.0020 (7)
C11	0.0258 (10)	0.0230 (9)	0.0175 (9)	−0.0092 (8)	−0.0005 (8)	0.0001 (7)
C2	0.0186 (9)	0.0172 (9)	0.0203 (10)	−0.0062 (7)	−0.0015 (7)	−0.0041 (7)
C16	0.0263 (10)	0.0228 (10)	0.0254 (11)	−0.0051 (8)	−0.0015 (8)	0.0019 (8)
C12	0.0279 (10)	0.0234 (10)	0.0218 (10)	−0.0054 (8)	−0.0050 (8)	0.0000 (8)
C5	0.0283 (10)	0.0296 (10)	0.0196 (10)	−0.0113 (8)	−0.0017 (8)	−0.0011 (8)
C6	0.0212 (9)	0.0188 (9)	0.0236 (10)	−0.0051 (7)	−0.0046 (8)	−0.0006 (7)
C4	0.0226 (9)	0.0330 (11)	0.0218 (10)	−0.0075 (8)	0.0037 (8)	−0.0095 (8)
C3	0.0199 (9)	0.0221 (9)	0.0264 (11)	−0.0017 (8)	−0.0007 (8)	−0.0073 (8)
C13	0.0368 (11)	0.0269 (10)	0.0230 (11)	−0.0055 (9)	−0.0085 (9)	0.0045 (8)
C17	0.0314 (11)	0.0389 (12)	0.0242 (11)	0.0010 (9)	−0.0037 (9)	0.0059 (9)
C14	0.0452 (13)	0.0362 (12)	0.0197 (11)	−0.0135 (10)	0.0027 (9)	0.0034 (9)
C15	0.0351 (11)	0.0326 (11)	0.0247 (11)	−0.0105 (9)	0.0052 (9)	0.0012 (9)
C18	0.0361 (12)	0.0306 (11)	0.0443 (14)	−0.0021 (9)	−0.0128 (10)	−0.0071 (10)
C20	0.0443 (13)	0.0514 (14)	0.0315 (13)	−0.0038 (11)	0.0129 (11)	0.0080 (11)
C19	0.0314 (13)	0.0332 (13)	0.128 (3)	0.0019 (11)	0.0098 (15)	0.0346 (15)

### Geometric parameters (Å, °)

**Table:**

O2—C9	1.224 (2)	C5—C6	1.386 (3)
O3—C11	1.382 (2)	C5—C4	1.395 (3)
O3—C10	1.418 (2)	C5—H5	0.9300
O1—C1	1.225 (2)	C6—H6	0.9300
N3—C9	1.360 (2)	C4—C3	1.391 (3)
N3—N2	1.367 (2)	C4—H4	0.9300
N3—H1	0.91 (2)	C3—H3	0.9300
N1—C1	1.359 (2)	C13—C14	1.380 (3)
N1—C2	1.405 (2)	C13—H13	0.9300
N1—H2	0.91 (2)	C17—C19	1.509 (3)
C8—N2	1.293 (2)	C17—C18	1.519 (3)
C8—C7	1.456 (3)	C17—H17	0.9800
C8—C1	1.516 (2)	C14—C15	1.385 (3)
C7—C6	1.385 (3)	C14—H14	0.9300
C7—C2	1.402 (2)	C15—C20	1.512 (3)
C9—C10	1.509 (3)	C18—H18A	0.9600
C10—H10A	0.9700	C18—H18B	0.9600
C10—H10B	0.9700	C18—H18C	0.9600
C11—C16	1.381 (3)	C20—H20A	0.9600
C11—C12	1.411 (3)	C20—H20B	0.9600
C2—C3	1.380 (3)	C20—H20C	0.9600
C16—C15	1.399 (3)	C19—H19A	0.9600
C16—H16	0.9300	C19—H19B	0.9600
C12—C13	1.383 (3)	C19—H19C	0.9600
C12—C17	1.510 (3)		
			
C11—O3—C10	119.11 (13)	C7—C6—H6	120.8
C9—N3—N2	118.88 (14)	C5—C6—H6	120.8
C9—N3—H1	120.7 (13)	C3—C4—C5	121.62 (17)
N2—N3—H1	120.4 (13)	C3—C4—H4	119.2
C1—N1—C2	111.67 (14)	C5—C4—H4	119.2
C1—N1—H2	125.7 (15)	C2—C3—C4	117.29 (16)
C2—N1—H2	122.5 (15)	C2—C3—H3	121.4
N2—C8—C7	125.50 (16)	C4—C3—H3	121.4
N2—C8—C1	128.37 (16)	C14—C13—C12	122.04 (18)
C7—C8—C1	106.13 (14)	C14—C13—H13	119.0
C8—N2—N3	116.19 (15)	C12—C13—H13	119.0
C6—C7—C2	120.41 (17)	C12—C17—C19	109.79 (19)
C6—C7—C8	132.69 (16)	C12—C17—C18	115.18 (18)
C2—C7—C8	106.90 (15)	C19—C17—C18	108.82 (18)
O2—C9—N3	123.63 (17)	C12—C17—H17	107.6
O2—C9—C10	121.00 (15)	C19—C17—H17	107.6
N3—C9—C10	115.35 (14)	C18—C17—H17	107.6
O3—C10—C9	109.16 (13)	C13—C14—C15	121.05 (19)
O3—C10—H10A	109.8	C13—C14—H14	119.5
C9—C10—H10A	109.8	C15—C14—H14	119.5
O3—C10—H10B	109.8	C14—C15—C16	118.21 (18)
C9—C10—H10B	109.8	C14—C15—C20	121.08 (18)
H10A—C10—H10B	108.3	C16—C15—C20	120.71 (18)
O1—C1—N1	127.57 (16)	C17—C18—H18A	109.5
O1—C1—C8	126.61 (15)	C17—C18—H18B	109.5
N1—C1—C8	105.81 (15)	H18A—C18—H18B	109.5
O3—C11—C16	123.51 (16)	C17—C18—H18C	109.5
O3—C11—C12	114.84 (16)	H18A—C18—H18C	109.5
C16—C11—C12	121.63 (17)	H18B—C18—H18C	109.5
C3—C2—C7	121.69 (17)	C15—C20—H20A	109.5
C3—C2—N1	128.83 (16)	C15—C20—H20B	109.5
C7—C2—N1	109.48 (15)	H20A—C20—H20B	109.5
C11—C16—C15	120.33 (17)	C15—C20—H20C	109.5
C11—C16—H16	119.8	H20A—C20—H20C	109.5
C15—C16—H16	119.8	H20B—C20—H20C	109.5
C13—C12—C11	116.72 (17)	C17—C19—H19A	109.5
C13—C12—C17	122.95 (17)	C17—C19—H19B	109.5
C11—C12—C17	120.32 (17)	H19A—C19—H19B	109.5
C6—C5—C4	120.49 (17)	C17—C19—H19C	109.5
C6—C5—H5	119.8	H19A—C19—H19C	109.5
C4—C5—H5	119.8	H19B—C19—H19C	109.5
C7—C6—C5	118.50 (16)		
			
C7—C8—N2—N3	−179.84 (15)	C1—N1—C2—C7	0.0 (2)
C1—C8—N2—N3	0.7 (3)	O3—C11—C16—C15	−179.07 (18)
C9—N3—N2—C8	179.15 (15)	C12—C11—C16—C15	−0.8 (3)
N2—C8—C7—C6	0.1 (3)	O3—C11—C12—C13	178.34 (17)
C1—C8—C7—C6	179.69 (18)	C16—C11—C12—C13	0.0 (3)
N2—C8—C7—C2	−179.00 (17)	O3—C11—C12—C17	−2.0 (3)
C1—C8—C7—C2	0.56 (18)	C16—C11—C12—C17	179.61 (19)
N2—N3—C9—O2	4.4 (2)	C2—C7—C6—C5	0.6 (3)
N2—N3—C9—C10	−174.08 (14)	C8—C7—C6—C5	−178.44 (18)
C11—O3—C10—C9	−178.08 (14)	C4—C5—C6—C7	−0.9 (3)
O2—C9—C10—O3	169.41 (15)	C6—C5—C4—C3	0.7 (3)
N3—C9—C10—O3	−12.1 (2)	C7—C2—C3—C4	−0.3 (3)
C2—N1—C1—O1	−178.51 (17)	N1—C2—C3—C4	179.26 (17)
C2—N1—C1—C8	0.39 (19)	C5—C4—C3—C2	−0.1 (3)
N2—C8—C1—O1	−2.1 (3)	C11—C12—C13—C14	0.7 (3)
C7—C8—C1—O1	178.33 (17)	C17—C12—C13—C14	−178.9 (2)
N2—C8—C1—N1	178.96 (17)	C13—C12—C17—C19	103.4 (2)
C7—C8—C1—N1	−0.59 (18)	C11—C12—C17—C19	−76.2 (3)
C10—O3—C11—C16	−3.0 (3)	C13—C12—C17—C18	−19.8 (3)
C10—O3—C11—C12	178.61 (16)	C11—C12—C17—C18	160.53 (18)
C6—C7—C2—C3	0.0 (3)	C12—C13—C14—C15	−0.5 (3)
C8—C7—C2—C3	179.27 (16)	C13—C14—C15—C16	−0.4 (3)
C6—C7—C2—N1	−179.60 (16)	C13—C14—C15—C20	179.1 (2)
C8—C7—C2—N1	−0.35 (19)	C11—C16—C15—C14	1.0 (3)
C1—N1—C2—C3	−179.62 (17)	C11—C16—C15—C20	−178.5 (2)

### Hydrogen-bond geometry (Å, °)

**Table:**

*D*—H···*A*	*D*—H	H···*A*	*D*···*A*	*D*—H···*A*
N3—H1···O1	0.91 (2)	2.06 (2)	2.768 (2)	134.1 (19)
N3—H1···O3	0.91 (2)	2.17 (2)	2.574 (2)	106.1 (17)
N1—H2···O2^i^	0.91 (2)	1.93 (2)	2.8174 (19)	164 (2)
C17—H17···O1	0.98	2.43	3.247 (2)	140

Symmetry codes: (i) *x*−1, *y*+1, *z*.
